# Blue News Update: BODIPY-GTP Binds to the Blue-Light Receptor YtvA While GTP Does Not

**DOI:** 10.1371/journal.pone.0029201

**Published:** 2012-01-11

**Authors:** Matthias Dorn, Marcel Jurk, Peter Schmieder

**Affiliations:** 1 Leibniz-Institut für Molekulare Pharmakologie, Berlin, Germany; 2 Freie Universität Berlin, Institute of Chemistry and Biochemistry, Berlin, Germany; University of Groningen, Netherlands

## Abstract

Light is an important environmental factor for almost all organisms. It is mainly used as an energy source but it is also a key factor for the regulation of multiple cellular functions. Light as the extracellular stimulus is thereby converted into an intracellular signal by photoreceptors that act as signal transducers. The blue-light receptor YtvA, a bacterial counterpart of plant phototropins, is involved in the stress response of *Bacillus subtilis*. The mechanism behind its activation, however, remains unknown. It was suggested based on fluorescence spectroscopic studies that YtvA function involves GTP binding and that this interaction is altered by absorption of light. We have investigated this interaction by several biophysical methods and show here using fluorescence spectroscopy, ITC titrations, and three NMR spectroscopic assays that while YtvA interacts with BODIPY-GTP as a fluorescent GTP analogue originally used for the detection of GTP binding, it does not bind GTP.

## Introduction

The detection of light is essential for nearly all organisms. It is not only a source of energy but also triggers the regulation of numerous biochemical pathways and physiological processes. Especially plants have developed a wide range of different photoreceptors to analyse environmental light by means of its intensity, direction, duration and color [1]. One class of photoreceptors are the phototropin related LOV proteins that are able to respond to blue light. They are abundant in a variety of different organisms reaching from prokaryotes to fungi and plants [2]–[4]. Common to all LOV proteins is the presence of at least one N-terminal LOV (light, oxygen, voltage) domain and a C-terminal effector domain linked to each other by a short linker peptide [5]–[8]. The LOV domain acts as a sensor for blue light harbouring a flavin molecule as the light-sensing chromophore. The effector domains of LOV proteins exhibit a wide range of different functions such as kinases, phosphatases or stress factor regulators [9].

YtvA of the common soil bacterium *Bacillus subtilis* belongs to the class of LOV proteins. It is a ∼60 kDa homodimer [10] and each subunit consists of a N-terminal LOV domain and a C-terminal sulphate transporter and anti sigma factor antagonist (STAS) domain linked to each other by a helical amino acid sequence, denoted Jα-helix. The protein is involved in the general stress response pathway of *Bacillus subtilis* [11]–[14] but its exact function remains unclear. The photocycle of YtvA is well understood [15] but the link between light induced formation of the chromophore adduct and activation of the effector domain is still missing. In contrast to LOV proteins undergoing substantial conformational changes upon illumination [16], [17], no large-scale structural changes were observed for YtvA [10]. Möglich *et al.* suggested a mechanism based on a rotational movement of the putative coiled-coil structured Jα-helices from both monomers [18]–[20]. This possibly alters the orientation of the STAS domains which subsequently leads to a change of binding properties to potential interaction partners. It has been suggested that an interaction partner for the STAS domain could be nucleoside triphosphates (NTPs) [21]. This assumption was originally based on sequence homology to other STAS domains, in particular to SPOIIAA from *Bacillus subtilis* or the STAS domain of Rv1739c from *Mycobacterium tuberculosis* that have both been shown to bind NTPs [22], [23]. Another hint for a potential GTP-binding capability of YtvA is the existence of two classical GTP-binding motifs, DXXG and NKXD, within its STAS domain (D^193^LSG and N^236^KLD). It has been shown that beside other conserved regions both motifs are jointly responsible for the interaction of GTP with G-proteins [24]. Further evidence for NTP binding of the STAS domain of YtvA was provided by Buttani *et al.* [25] that used a fluorescence assay to investigate the binding of GTP to YtvA and found a binding constant of K_D_ = 38 µM for illuminated YtvA and a increased affinity after dark reversion. This assay has since been used to study the effect of mutations on the activation mechanism in YtvA [25]–[28]. More recently, however, Nakasone *et al.* could not confirm that GTP binds to YtvA but found that the fluorescence labeled GTP analog (BODIPY-GTP) used in the assays binds unspecifically to YtvA [29]. The aim of the investigations described here was originally to determine the precise binding site for GTP on YtvA using heteronuclear NMR. After a repeated failure to detect any binding we used fluorescence spectroscopy, isothermal titration calorimetry (ITC) and NMR binding assays (protein as well as ligand detected) to investigate not only the binding of GTP to YtvA and to the isolated STAS domain of YtvA (further on denoted YtvA-STAS) but also that of the fluorescent analogue, BODIPY-GTP. We can show that while BODIPY-GTP does in fact bind to YtvA via the fluorescent dye in an unspecific manner, GTP does not show any binding to either protein and that thus YtvA function does not involve GTP binding.

## Results and Discussion

**Figure 1:**
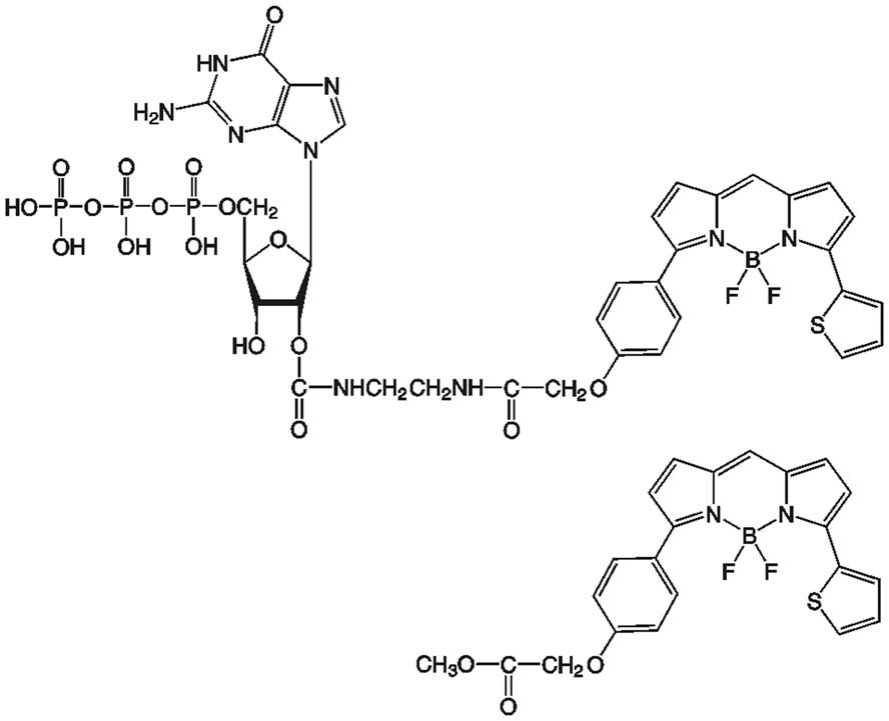
Fluorescent dyes. Structural formula of BODIPY-GTP (top) and BODIPY methyl ester (bottom).

To investigate the binding of GTP to YtvA we applied three different biophysical methods (Fluorescence spectroscopy, ITC, NMR) to the full length protein and the isolated STAS domain. To be able to separate the effects of GTP and the fluorescent dye, three small molecules were used: GTP, BODIPY-GTP and BODIPY alone, the latter two are shown in Figure 1.

### Fluorescence spectroscopy

**Figure 2:**
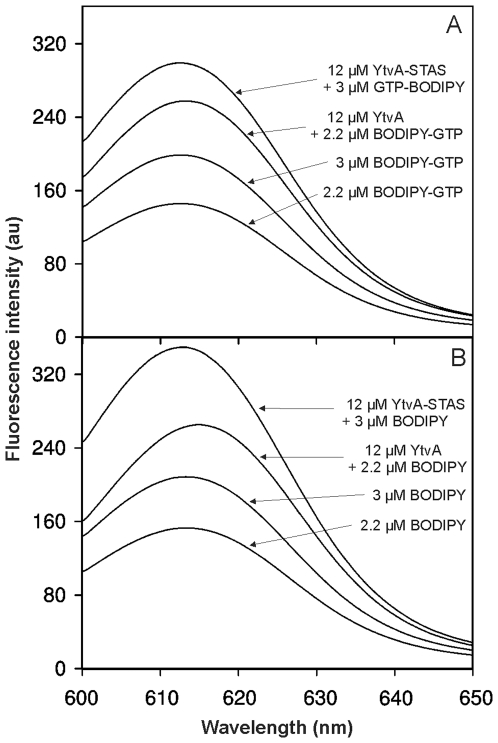
Comparison of fluorescence spectra. Fluorescence spectra obtained from experiments using BODIPY-GTP (A) or BODIPY (B) as the fluorophore. The spectra are shown in a ‘bottom to the top’ order for 2.2 µM sole fluorophore, 3 µM sole fluorophore, 2.2 µM fluorophore with 12 µM YtvA and 3 µM fluorophore with 12 µM YtvA-STAS.

As a first step of the investigation we repeated the fluorescence experiments of Buttani *et al.* [25]. The fluorescence spectroscopy experiments clearly showed the binding of BODIPY-GTP to YtvA and YtvA-STAS (Fig. 2A), respectively. After addition of YtvA or YtvA-STAS to the fluorophore solution the relative fluorescence was significantly increased. This is in agreement with the results presented in the literature so far [25], [29]. Since BODIPY is very hydrophobic, however, interactions of BODIPY with hydrophobic regions on the protein surface could be the reason for binding. Such unspecific interactions would also lead to a fluorescence enhancement and would therefore give a false positive result, making the detection of effects of an interaction of GTP alone nearly impossible. We therefore repeated the fluorescence spectroscopic experiments with BODIPY alone, the spectra are shown in Figure 2B. The relative fluorescence intensity changes were comparable to those obtained from the BODIPY-GTP experiment. This suggests that only the fluorophore is binding to the protein but the GTP part does not. The latter, however, can not be excluded since a weak GTP binding capability could simply be masked due to the large fluorescence enhancement resulting from fluorophore binding.

### Isothermal titration spectroscopy

We therefore performed ITC experiments with YtvA and YtvA-STAS. An advantage of the method is that beside BODIPY-GTP also GTP alone can be used for the experiments. The isolated dye alone is not soluble enough in buffer solution to perform ITC experiments. All calculated values from the ITC measurements are shown in Table 1 and the titration curves are given in Figure S1 and Figure S2 in the supplementary material. An interaction of GTP with either YtvA or YtvA-STAS was not observable, only a small heat resulting from dilution effects was detectable. The titration of YtvA with BODIPY-GTP, however, produced a typical binding curve and a calculated dissociation constant of 2.5 µM. Data obtained from the titration of YtvA-STAS with BODIPY-GTP were analysed using the software Sedphat v9.01 [30] because the titration did not lead to a complete saturation. The quality of the data from the latter titration was therefore not as high as for YtvA, the lower temperature, but also the poor stability of YtvA-STAS could be reasons for that. However, Sedphat v9.01 is able to handle such difficulties much better than the Microcal Origin software and produced a well fitted binding curve (see Figure S3). The best-fit values for the thermodynamic parameters were K_D_ = 7.6 µM and ΔH = −48 kcal/mol. Whereas the ΔH value was associated with great uncertainty, K_D_ was defined precisely, as demonstrated by variation of the reduced χ^2^ around the best-fit minimum [31], [32]. In the latter procedure, K_D_ is frozen at values close to but different from its best-fit value, and the fit is repeated by optimising the other parameters (i.e., ΔH, the heat of dilution and the incompetent fraction of BODIPY-GTP in the syringe). This provides a projection of the reduced χ^2^ value onto the K_D_ plane (see Figure S4, blue circles). The reduced χ^2^ threshold for the 99% confidence interval was then calculated on the basis of Fisher's F distribution (see Figure S4, red line). The 99% confidence interval for K_D_ thus determined ranged from 6.2 µM to 9.4 µM. The ITC data confirmed the results from the fluorescence spectroscopy experiments indicating that only the fluorophore binds while GTP does not. Additionally the data suggest that the STAS domain is responsible for the interaction. Interestingly, we found a significant difference in the stoichiometry between the reaction of BODIPY-GTP with YtvA and YtvA-STAS. While YtvA-STAS binds BODIPY-GTP in a 1∶1 ratio, the ratio for YtvA complexed with BODIPY-GTP is only 2∶1. While YtvA is clearly dimeric under the tested conditions [10], sedimentation velocity experiments with YtvA-STAS performed in our lab showed that this domain is mostly monomeric at low concentrations (<100 µM) but tends to dimerize with increasing concentration [33]. Furthermore, YtvA-STAS is only stable below 12°C and starts to aggregate at physiological temperatures, while the full length protein exhibits a much higher stability. We therefore assume that YtvA-STAS possesses hydrophobic regions on its surface and that these regions are covered to some extend in dimeric full length YtvA. Since the binding of BODIPY-GTP seems to be driven by hydrophobic interactions this could explain the above mentioned differences of the stoichiometric ratios.

**Table 1:** Results of the ITC experiments with YtvA and YtvA-STAS.

protein	ligand	ΔH (kcal/mol)	-TΔS (kcal/mol)	N	K_D_ (µM)	ΔG° (kcal/mol)
YtvA-STAS	GTP	/	/	/	n. b. d.	/
YtvA-STAS	BODIPY-GTP	−48	41.3	0.96	7.6	−6.7
YtvA	GTP	/	/	/	n. b. d.	/
YtvA	BODIPY-GTP	−9.6±0.3	2.0	0.54±0.01	2.5±0.3	−7.6

n.b.d.: no binding detected.

### NMR spectroscopy

NMR spectroscopy is well established as a method for the detection of interactions between proteins and small ligands, in particular if the interaction is weak. Two types of experiments are possible: using ^15^N-labeled protein the signals of the protein can be observed and changes upon binding detected by recording two-dimensional ^1^H,^15^N correlations. In addition, ligand-detected experiments can be performed by recording STD [34] or WaterLOGSY [35], [36] experiments in the presence and absence of unlabeled protein. Both proteins were available in ^15^N-labeled form, for the full length protein an assignment of the ^1^H,^15^N correlation (for the dark state) was available as well [33].

**Figure 3:**
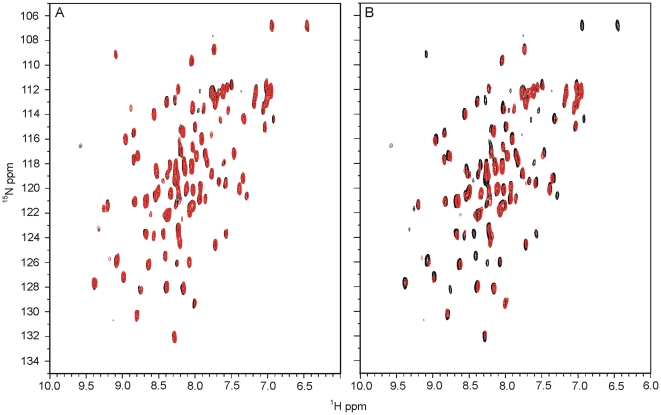
Superposition of ^1^H-^15^N-HSQC spectra of YtvA-STAS without and with added ligands. ^1^H-^15^N-HSQC spectra of (A) 30 µM uniformly ^15^N-labeled YtvA-STAS without (black) and with 180 µM GTP (red) and (B) 30 µM uniformly ^15^N-labeled YtvA-STAS without (black) and with 180 µM BODIPY-GTP (red).

Two-dimensional ^1^H,^15^N correlation spectra (^1^H,^15^N-HSQCs) were recorded for YtvA-STAS with and without the addition of an 6-fold excess of GTP and are shown in Figure 3A. No significant changes of chemical shift or intensities were observable in these experiments, indicating that no interaction occurs. In contrast, the overlay of ^1^H-^15^N-HSQC spectra of YtvA-STAS alone and YtvA-STAS treated with BODIPY-GTP shows large intensity changes for various cross peaks (Fig. 3B). This confirmed the interaction between both species and showed that BODIPY-GTP is in intermediate exchange with respect to the NMR time scale leading to line broadening of those protein signals from residues participating in the binding.

**Figure 4:**
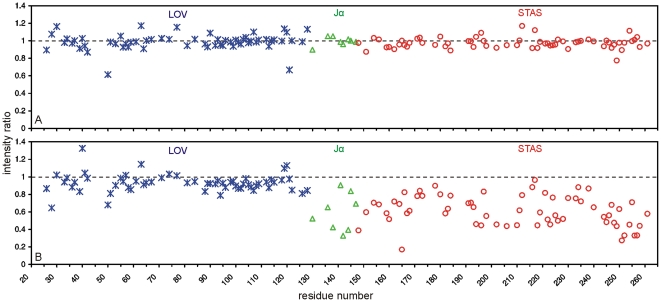
Plots of ^1^H-^15^N-TROSY cross peak intensity ratios of YtvA. Plots of the ^1^H-^15^N-TROSY cross peak intensity ratios of (A) 50 µM uniformly ^2^H-^15^N-labeled YtvA with and without 500 µM GTP and (B) 50 µM uniformly ^2^H-^15^N-labeled YtvA with and without 500 µM BODIPY-GTP against the YtvA sequence. Corresponding 2D ^1^H-^15^N-TROSY spectra were recorded with YtvA kept in the dark state. Residues are represented by asterisks for the LOV domain, triangles for the Jα-helix and circles for STAS domain. An intensity ratio of 1 (dashed line) means that the addition of the appropriate ligand has shown no effect on corresponding residues.

We repeated these experiments with YtvA, except that a 10-fold excess of the appropriate ligand was added and that – given the size of the protein - ^1^H,^15^N correlations were recorded as ^1^H-^15^N-TROSY spectra [37], [38]. Consistent with the results obtained from YtvA-STAS no significant differences between the ^1^H-^15^N-TROSY spectra of YtvA without and with GTP was observed but various changes of cross peak intensities were observed in presence of BODIPY-GTP. A superposition of the spectra from both experiments is given in Figure S5 of the supplementary material. We quantified the effects of the ligands for those residues that show well resolved distinct single peaks. Figure 4 shows plots of the ratios of signal intensity against the corresponding residues of the YtvA sequence for GTP (Figure 4A) and BODIPY-GTP (Figure 4B). In case of GTP the intensity ratios fluctuate closely around 1 indicating that there are nearly no differences between the corresponding spectra. Especially residues of the D^193^LSG and the N^236^KLD motif were not influenced by GTP. In contrast, the binding of BODIPY-GTP resulted in significant effects on YtvA. While its LOV domain is nearly unaffected and the average ratio of intensity within the core domain (amino acids 26–128) added up to 0.93+/−0.1, the YtvA-STAS domain and also the Jα-helix were strongly affected. Average intensity change ratios were 0.60+/−0.21 for Jα and 0.61+/−0.17 for YtvA-STAS, respectively.

Thus the binding of BODIPY-GTP appears to be confined to the Jα-helix and the STAS domain but in a rather unspecific manner. A similarily unspecific mechanism for the interaction of BODIPY-GTP with hydrophobic regions of the LOV domain of YtvA was suggested by Nakasone *et al.* [29], their assumption being based on the comparison of BODIPY-GTP binding to different truncated versions of the LOV domain part of the protein. Such truncations may alter the hydrophobicity of the resulting surface leading to interactions with hydrophobic ligands which may explain why binding to the LOV domain was detected while the interaction seems to be confined to the STAS domain and the Jα-helix in our experiments. As indicated in the ITC experiments we detect a similar effect when truncating the protein: the binding of BODIPY-GTP to YtvA-STAS differs from that to YtvA. We know from further NMR experiments performed with full length YtvA [33] that the Jα-helix and the STAS domain are more flexible than the LOV domain. This could explain why the two former parts of the protein are more accessible to unspecific hydrophobic interactions while the LOV domain - being a relatively rigid and tight binding dimer - does not expose any hydrophobic patches as easily as the linker and the STAS domain during their movements. More importantly, however, the NMR experiments with protein detection described above indicate again that no binding of GTP to YtvA is taking place.

**Figure 5:**
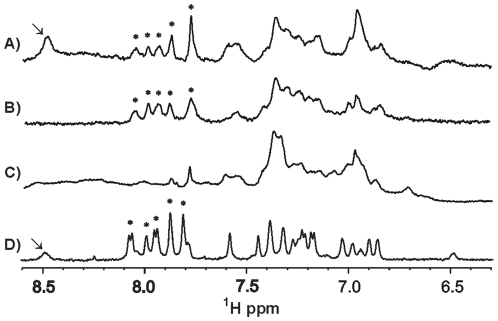
Sections of ligand detected 1D ^1^H-NMR spectra from the analysis of weak molecular interactions between dark state YtvA and BODIPY-GTP. WaterLOGSY (A) and STD-Watergate (B) of 20 µM YtvA mixed with 200 µM BODIPY-GTP. Watergate of (C) 20 µM dark state YtvA and (D) 200 µM BODIPY-GTP. Signals of protons from the aromatic BODIPY part are marked by asterisks. Exchangeable ligand protons are marked by arrows.

To confirm the results obtained with detection of the protein and to investigate a possible interaction of GTP with light-activated YtvA we applied two further NMR techniques; the STD [34] and the WaterLOGSY [35], [36] experiment. Both techniques are based on the detection of the ligand and detect changes in its NMR spectrum caused by an interaction with the protein. While in a STD spectrum binding is indicated by the presence of signals from the ligand, it is detectable in a WaterLOGSY experiment by a sign change of the signals from the small molecule. Given that latter technique relies on exchange with the bulk solvent, hydrogens of the ligand that are in exchange with it will always appear irrespective of binding. We first performed the experiments using BODIPY-GTP and its interaction with YtvA could be clearly verified. Figure 5 shows the expanded regions of the NMR spectra, the WaterLOGSY and the STD experiment of YtvA complexed with BODIPY-GTP are shown in Figure 5A and Figure 5B, respectively. Also displayed are the reference spectra of YtvA (Figure 5C) and BODIPY-GTP (Figure 5D). Signals belonging to the aromatic protons from the BODIPY part are highlighted by asterisks. The signal marked by an arrow resulted from an exchangeable proton of BODIPY-GTP and is therefore only visible in the reference and the WaterLOGSY spectrum. Both types of experiments confirmed that the hydrophobic part of BODIPY is responsible for the interaction. The repetition of the experiments with YtvA in the lit-state (continuously illuminated) produced comparable results (data not shown).

**Figure 6:**
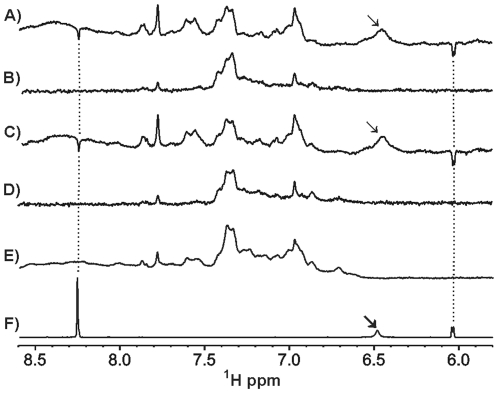
Sections of ligand detected 1D ^1^H-NMR spectra from the analysis of weak molecular interactions between YtvA and GTP. WaterLOGSY (A) and STD-Watergate (B) of 20 µM dark state YtvA mixed with 200 µM GTP. WaterLOGSY (C) and STD-Watergate (D) of 20 µM lit state YtvA mixed with 200 µM GTP. Watergate of (E) 20 µM dark state YtvA and (F) 200 µM GTP. Signals belonging to GTP are connected by dotted lines. Exchangeable ligand protons are marked by arrows.

To study the interaction between GTP and YtvA all experiments were repeated under the same conditions as previously used with BODIPY-GTP. Regions of the 1D ^1^H -NMR spectra are given in Figure 6. A 1D ^1^H-NMR reference spectrum of YtvA is only shown for the dark state (Fig. 6E) since the difference to the lit spectrum is rather small. Figure 5F shows the reference spectrum of GTP. The WaterLOGSY and STD spectra of GTP in the presence of dark state YtvA are shown in Figure 6A and Figure 6B, respectively. The corresponding spectra achieved from the experiment performed under continuous illumination are shown in Fig. 6C (WaterLOGSY) and Fig. 6D (STD). The signal marked by an arrow belongs to a water exchangable proton of GTP. The sharp signal at 8.25 ppm in the GTP reference originates from the proton bound to C8 of the purine and the doublet at 6.04 ppm is derived from H1′ of the ribose. Both GTP signals are absent in the STD spectra and negative in the WaterLOGSY spectra. This again shows unambiguously that YtvA, independent of its activation state, does not interact with GTP.

In conclusion we have shown using several biophysical techniques that while the fluorescent analogue of GTP, BODIPY-GTP, does indeed interact with YtvA in an unspecific manner, GTP itself does not and that these effects are independent of the presence of blue light. YtvA therefore appears to be no GTP-binding protein and experimental results based on the assumption of an interaction between GTP and YtvA have to be reconsidered. That also applies to theories assuming a GTP dependent function or activation mechanism of YtvA, and the interpretation of in vivo mutational studies [25]–[28].

## Materials and Methods

### Buffer

Buffer A: 30 mM Tris-HCl pH = 7.9; 300 mM NaCl; 1 mM MgSO_4_; 5 mM imidazole; 10% glycerol; Complete® protease inhibitor mix (Roche)

Buffer B: 20 mM Tris-HCl pH = 7.5; 300 mM NaCl; 1 mM 2-Mercaptoethanol

Buffer C: 10 mM Na2HPO4; 1.8 mM KH2PO4; 2.7 mM KCl; 137 mM NaCl; 5 mM 2-Mercaptoethanol; pH = 7.4

Buffer D: 20 mM Tris-HCl pH = 7.4; 50 mM NaCl; 100 µM TCEP

Buffer E: 20 mM Na-Phosphate pH = 7.5, 150 mM NaCl, 2 mM DTT

### Cloning

Construction of the expression plasmids for full-length YtvA and for the STAS domain of YtvA (amino acids 148–261) was performed as described earlier [10]. For cloning of the STAS domain the following primers were used:

Forward tobacco etch virus (TEV): 5′-gac gac gac aag atg gaa aac ctg tat ttc cag-3′

Forward YtvA-STAS: 5′-aac ctg tat ttc cag gga act cct att gtc ccg att cg-3′

Reverse YtvA-STAS: 5′-gag gag aag ccc ggt tta cat aat cgg aag cac ttt aac g-3′

Via the PCR, a TEV cleavage site was introduced, leaving an additional Gly at the the N-terminus after proteolysis. In YtvA the wild-type Met^1^ was replaced by this glycine. The plasmid described earlier by Losi *et al.* [39] was used as a template. The amplified DNA sequences were cloned into the pET 30 EK/LIC vector using the ligation independent cloning technique (EK/LIC system, Merck KGaA) [40]. All clones were checked by bidirectional DNA sequence analysis (Invitek, Berlin, Germany).

### Protein expression and purification

Expression and purification of unlabeled YtvA was done following the protocol of Jurk *et al.* [10]. Recombinant expression of YtvA-STAS was also carried out using T7-Express Rosetta2 cells as expression host (New England Biolabs GmbH, Frankfurt a.M., Germany). For unlabeled YtvA-STAS cells were grown in TB medium supplemented with 2% glucose and the appropriate antibiotics. Starting from an overnight pre-culture fresh media was inoculated to an OD_600_ of 0.15 and cells were grown at 37°C to a final OD_600_ of 1.0–1.2. Cultures were cooled down to 22°C and protein expression was induced by adding IPTG (Carl Roth GmbH, Karlsruhe, Germany) to 1 mM final concentration. 18–20 h after induction cells were harvested by centrifugation in a pre-chilled rotor at 4°C and either stored at −80°C or, after resuspension in buffer A, directly lysed with an EmulsiFlex-C3 High-Pressure Homogenizer (Avestin Inc., Ottawa, Canada). ^15^N-labeled YtvA-STAS was expressed in M9 minimal medium [41], supplemented with twice the concentration of M9-salt, using a high cell density fermentation system (FedBatch Pro, DASGIP, Germany). For details see Fiedler *et al.* [42]. A pre-culture of 400 ml TB (supplemented as described above) was inoculated from an overnight culture to an OD_600_ of 0.05 and cells were grown at 37°C to a final OD_600_ between 1.0–1.5. Cells were harvested by mild centrifugation (1000×g, 10 min) at room temperature and the pellet was resuspended in 50 ml M9 minimal medium. 25 ml of the resuspended cells were used for the inoculation of one 250 ml bioreactor. During the batch phase the temperature was kept constantly at 37°C. The pH of the media was kept above 7 during the whole fermentation process by stepwise addition of 1 M NaOH. After depletion of mostly all C and N sources the bioreactor was cooled to 22°C and 15 ml of the ^15^N-labeled expression feed was pumped into the bioreactor with a flow rate of 1.3 ml/h. The induction was started 30 min afterwards by the addition of IPTG to a final concentration of 1 mM. 1 h after complete addition of the expression feed cells were harvested or directly lysed as described above. Production of ^2^H-^15^N-labeled YtvA was performed as described in Jurk *et al.* [33]. Unlabeled and ^15^N-labeled YtvA-STAS were purified by affinity chromatography on a Poros® 20 mc column (Workstation Vision, Applied Biosystems) using recommended standard buffers. TEV cleavage was performed during dialysis overnight at 8°C in Buffer B by addition of 1 OD_280_ TEV protease to 100 OD_280_ of fusion protein followed by a second affinity chromatography purification step to remove the TEV protease and the cleaved tag. TEV protease was recombinantly expressed as N-terminal His_6_-tagged fusion protein using *E. coli* BL21-DE3 as expression host. TEV protease clone was generously provided by Gunter Stier (EMBL, Heidelberg, Germany). Finally, the proteins were further purified by size exclusion chromatography using a 26/60 Superdex 75 column on a Pharmacia Biotech FPLC system (LKB GP-10) equilibrated with buffer C. Sample concentration was increased with a stirred Cell 8050 (Millipore GmbH, Schwalbach, Germany) equipped with Ultrafiltration Disc YM-10 (10 kDa NMWL) for Ytva or PLBC (3 kDa NMWL) for YtvA-STAS. Proteins were checked to be >95% pure by overloading a SDS-gel. Photochemical activity of YtvA was checked by comparison of UV-Vis spectra recorded from dark and illuminated samples.

### Sample preparation

All proteins used for fluorescence spectroscopy and 1D ^1^H-NMR experiments (STD/WaterLOGSY) were dialysed for 24 h against 1000-fold excess of buffer C at 8°C. Proteins used for Isothermal Titration Calorimetry were dialysed for 24 h at 8°C against 1000-fold excess of buffer D except for YtvA-STAS where the pH was set to 7.5 and 150 mM NaCl was used. 2D heteronuclear NMR experiments were done in buffer C for YtvA and buffer E for YtvA-STAS, respectively. Concentration and chromophore content of YtvA was determined according to Jurk *et al.* [10]. Concentration of YtvA-STAS was calculated from the absorption at 280 nm using a calculated molar extinction coefficient of 2980 M^−1^ cm^−1^ at 280 nm (ProtParam). GTP, BODIPY-GTP (BODIPY® TR GTP) and BODIPY (BODIPY® TR methyl ester), all purchased from Invitrogen, were not dialysed because of its small molecular weight, the tendency of BODIPY-GTP to adsorb to the dialysis membrane and the small solubility of BODIPY in aqueous solutions. Desired concentrations were achieved by dilution with the appropriate dialysis buffer.

### Fluorescence spectroscopy

Fluorescence spectroscopy was performed on a Jasco FP-6500 spectrofluorometer at 20°C using a 700 µl, 1 cm light-path half-micro fluorescence cuvette. Excitation wavelength was set to 590 nm with 5 nm slit width and emission was detected between 600–650 nm with low sensitivity. Reference spectra of the single components were recorded under the same conditions as used for the binding experiments (12 µM YtvA, 2.2 µM ligand and 12 µM STAS, 3 µM ligand). That includes the presence of 0.06% DMSO in all samples, since BODIPY is only available diluted in DMSO. The differences of the ligand concentrations resulted from the fact that the concentration of the YtvA stock solution was not as high as the concentration of the YtvA-STAS stock solution. All experiments were directly performed inside the cuvette. The appropriate components were pooled together, mixed by several inversion steps and measured after one minute of incubation. All measurements were performed twice. To prevent YtvA from photo-activation all experiments were done under safe red light (λ>620 nm).

### Isothermal Titration Calorimetry

Isothermal Titration Calorimetry experiments were performed with a VP-ITC microcalorimeter (Microcal®) at 25°C for YtvA and 12°C for YtvA-STAS, respectively. All titrations were performed twice except the titration of YtvA-STAS with BODIPY-GTP. After the dialysis the protein and ligand samples were diluted with the appropriate dialysis buffer. The 500 µM ligand solution was titrated into the sample cell containing either 60 µM YtvA, 30 µM YtvA or 50 µM YtvA-STAS except the titrations of YtvA-STAS with GTP where 1 mM GTP was titrated to 100 µM YtvA-STAS. The volume of each injection was 10 µl except the first one where only 2 µl were injected. Spacing time between each injection was 5 min. Control experiments were performed by an identical injection pattern of ligand into the sample cell containing only buffer to monitor dilution effects. Data analysis was performed using the Microcal Origin software. Baseline correction and peak integration were done manually. The association constant (K), standard enthalpy change (ΔH), standard entropy change (ΔS) and stoichiometry (N) for the interactions were obtained from fitting of the experimental titration curve. The fit was performed for a single binding site. The enthalpy, the association constant and the stoichiometry were kept variable during the fitting procedure. The data obtained from the titration of YtvA-STAS with BODIPY-GTP were calculated using the software Sedphat v9.01 [30]. Its algorithm fits data of titrations where a complete saturation could not be reached much better than the Microcal Origin software. Free enthalpy (ΔG°) was calculated from the fitted parameters using the following equation: ΔG° = -RTlnK = ΔH -TΔS.

### NMR experiments

NMR experiments were performed at 300 K on a Bruker Avance 600 MHz spectrometer equipped with a 5 mm triple resonance PFG (z-axis) cryo probe head. All spectra were processed using Topspin® 2.1 software. Analysis of crosspeak intensities from 2D heteronuclear NMR spectra was done using ccpnmr analysis v.2.1 software [43]. Heteronuclear NMR spectroscopic investigation of YtvA-STAS interaction with GTP/BODIPY-GTP was achieved by recording 2D ^1^H-^15^N-HSQC experiments. 30 µM uniformly ^15^N-labeled YtvA-STAS was treated either with 180 µM GTP or 180 µM BODIPY-GTP. Investigation of interactions between YtvA (dark state) and GTP or BODIPY-GTP was done by recording 2D ^1^H-^15^N-TROSY spectra of 50 µM uniformly ^2^H-^15^N-labeled YtvA with and without a 10-fold excess of the appropriate ligand. Additionally for YtvA, NMR spectroscopic investigations of weak molecular interactions were performed using WaterLOGSY [35], [36] and Saturation Transfer Difference (STD) experiments [34]. Both types of experiments were also applied to investigate the binding capabilities of the ligands to illuminated YtvA. For this purpose we used a Fiber Coupled Light Source that allows a continuous irradiation of the sample with λ_max_ = 455 nm directly within the magnet (for details see Figure S6 and S7 in the supplementary material). To achieve an uniform illumination of the NMR samples the end of the optical fiber was modified as described by Kuprov *et al.* [44]. Ligand detected experiments were performed with 20 µM YtvA plus a 10-fold excess of the appropriate ligand. 1D ^1^H -NMR spectra with water suppression (Watergate) [45] were recorded with the sole ligands and sole YtvA for reference, respectively. Concentrations were kept equal in all corresponding experiments. A reference spectrum (Watergate), a 1D WaterLOGSY and a 1D STD-Watergate spectrum was recorded during each interaction study.

## Supporting Information

Figure S1 **ITC titration curves of YtvA-STAS.** (A) 1 mM GTP in buffer, (B) 1 mM GTP in 100 µM YtvA-STAS and (C) 500 µM BODIPY-GTP in 50 µM YtvA-STAS. All titrations were performed at 12°C.
(EPS)

Figure S2 **ITC titration curves of dark state YtvA.** (A) 500 µM BODIPY-GTP in 60 µM YtvA, (B) 500 µM BODIPY-GTP in 30 µM YtvA and (C) 500 µM GTP in 60 µM YtvA. All titrations were performed at 25°C.
(EPS)

Figure S3 **Fit curve of the data obtained from the titration of YtvA-STAS with BODIPY-GTP.** The binding curve and the residuals were calculated with Sedphat v9.01.
(TIF)

Figure S4 **Plot of the confidence intervall analysis of the K_D_ obtained from the titration of YtvA-STAS with BODIPY-GTP.** The projection of the reduced χ^2^ values onto the K_D_ plane is shown by blue circles. The reduced χ^2^ threshold for the 99% confidence interval is shown by the red line. The 99% confidence interval for K_D_ (all values on or below the red line) ranges from 6.2 µM to 9.4 µM.
(TIF)

Figure S5 **Superposition of ^1^H-^15^N-TROSY spectra of dark state YtvA without and with added ligands.** ^1^H-^15^N-TROSY spectra of (A) 50 µM uniformly ^2^H-^15^N-labeled YtvA without (black) and with 500 µM GTP (red) and (B) 50 µM uniformly ^2^H-^15^N-labeled YtvA without (black) and with 500 µM BODIPY-GTP (red). All spectra were recorded with YtvA kept in the dark state.
(TIF)

Figure S6 **LED based Fiber Coupled Light Source for NMR sample illumination.** The light of a modified high power LED (Luxeon LXHL-LR5C, λ_max_ = 455 nm) is butt coupled into an optical fiber (Thorlabs 0,48NA multimode fiber BFH48-1000) and directly transmitted into a 5 mm NMR sample tube. The optical output power is adjustable by means of its intensity and duty cycle from 0 mW up to 8 mW (measured with a S302C Thermal Power Head, Thorlabs).
(JPG)

Figure S7 **NMR sample illumination with our Fiber Coupled Light Source.** Shown is the illumination of YtvA (500 µM in PBS) at λ_max_ = 455 nm directly inside the NMR tube. To achieve a homogeneous illumination within the hole sample the end of the fiber was etched stepwise (12 steps, 3 mm per step) in a mixture containing 30% hydrofluoric acid and 20% sulfuric acid at 60°C as described earlier by Kuprov *et al.* [44].
(JPG)
