# Supplementary figures and images for: Blue News Update: BODIPY-GTP Binds to the Blue-Light Receptor YtvA While GTP Does Not
Source: PLoS One. 2012 Jan 11;7(1):e29201. doi: 10.1371/journal.pone.0029201 (PMC3256143; doi:10.1371/journal.pone.0029201)

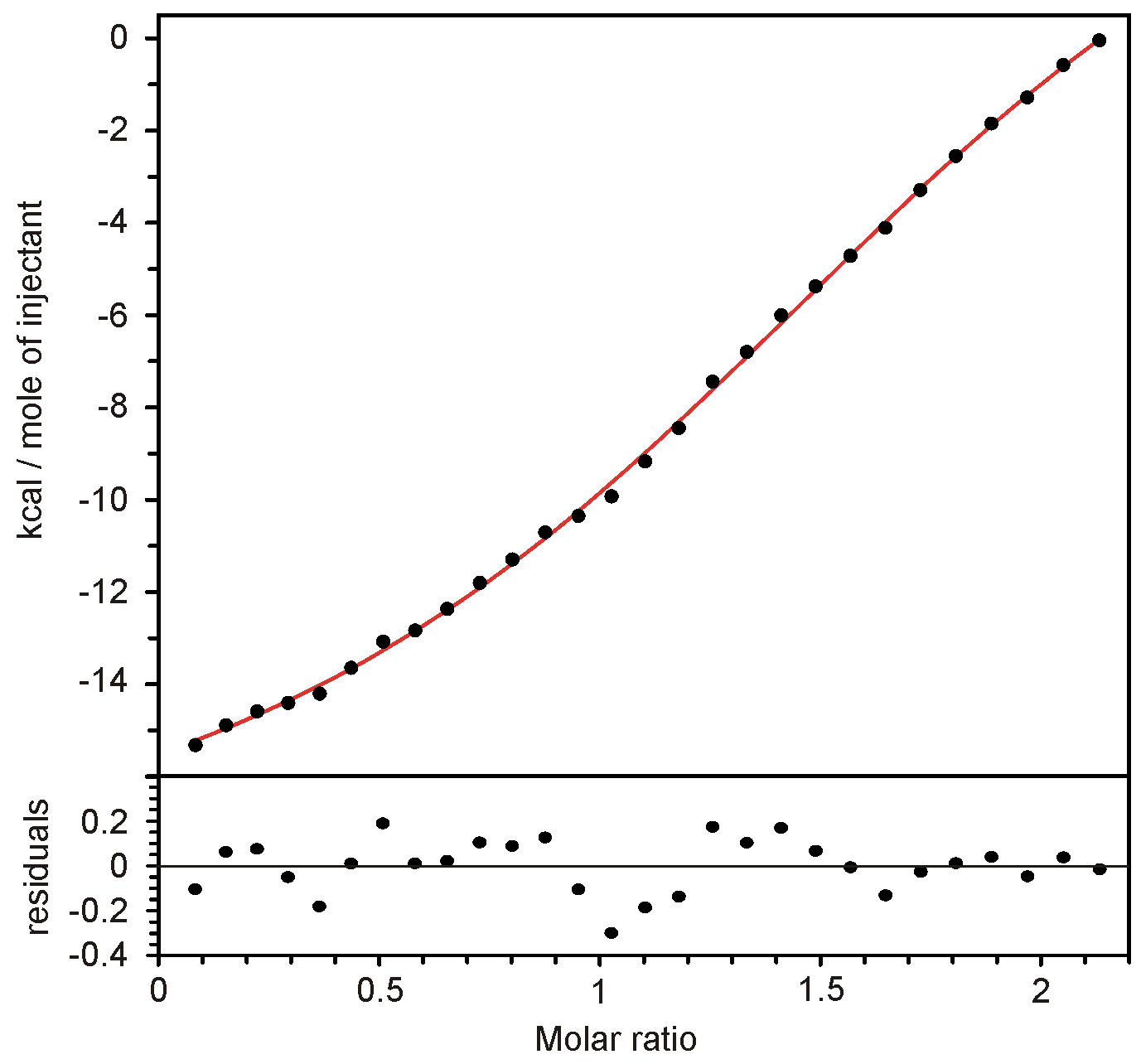

Supplement: Figure S3 — Fit curve of the data obtained from the titration of YtvA-STAS with BODIPY-GTP. The binding curve and the residuals were calculated with Sedphat v9.01. (TIF) [file pone.0029201.s003.tif]

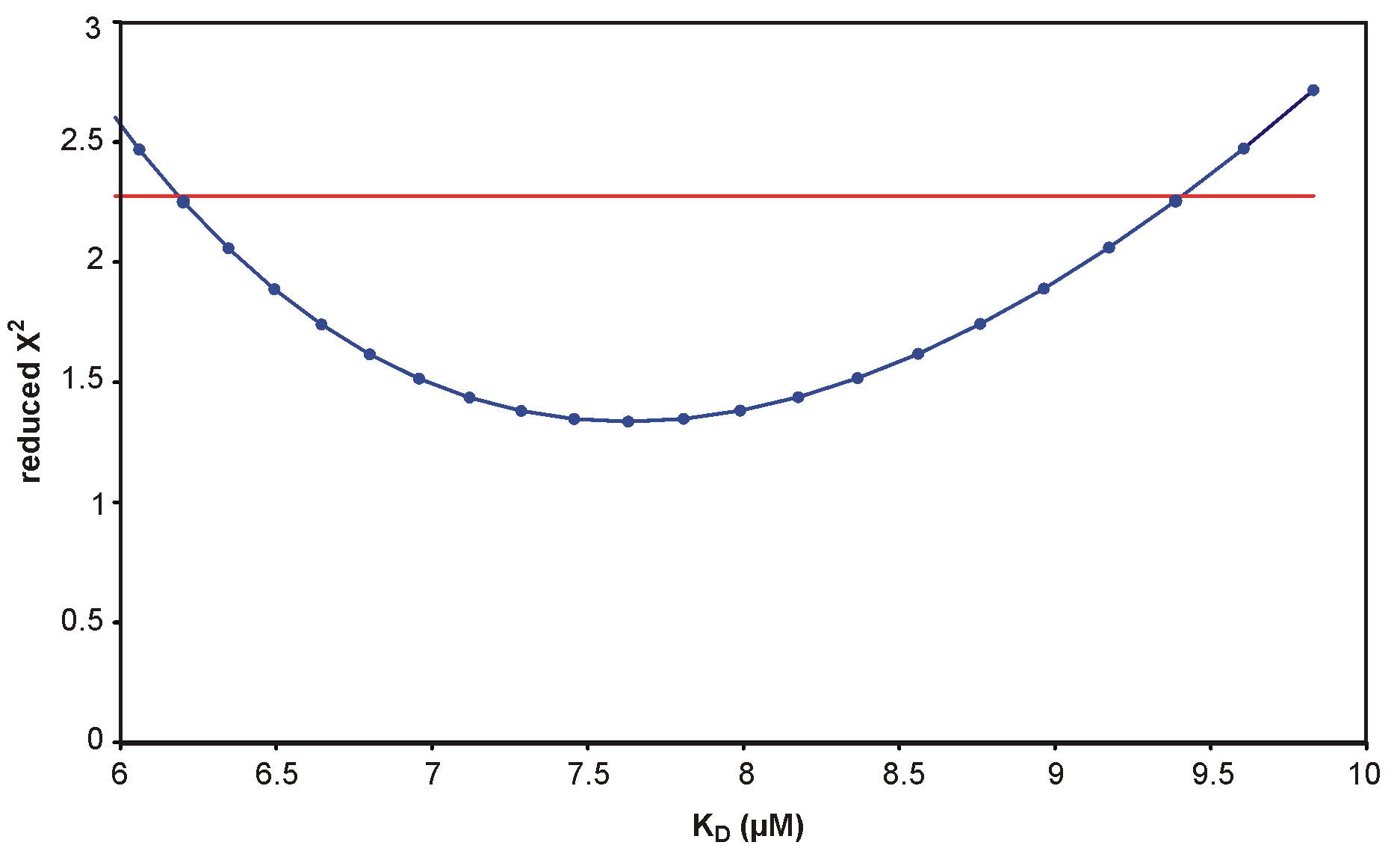

Supplement: Figure S4 — Plot of the confidence intervall analysis of the KD obtained from the titration of YtvA-STAS with BODIPY-GTP. The projection of the reduced χ2 values onto the KD plane is shown by blue circles. The reduced χ2 threshold for the 99% confidence interval is shown by the red line. The 99% confidence interval for KD (all values on or below the red line) ranges from 6.2 µM to 9.4 µM. (TIF) [file pone.0029201.s004.tif]

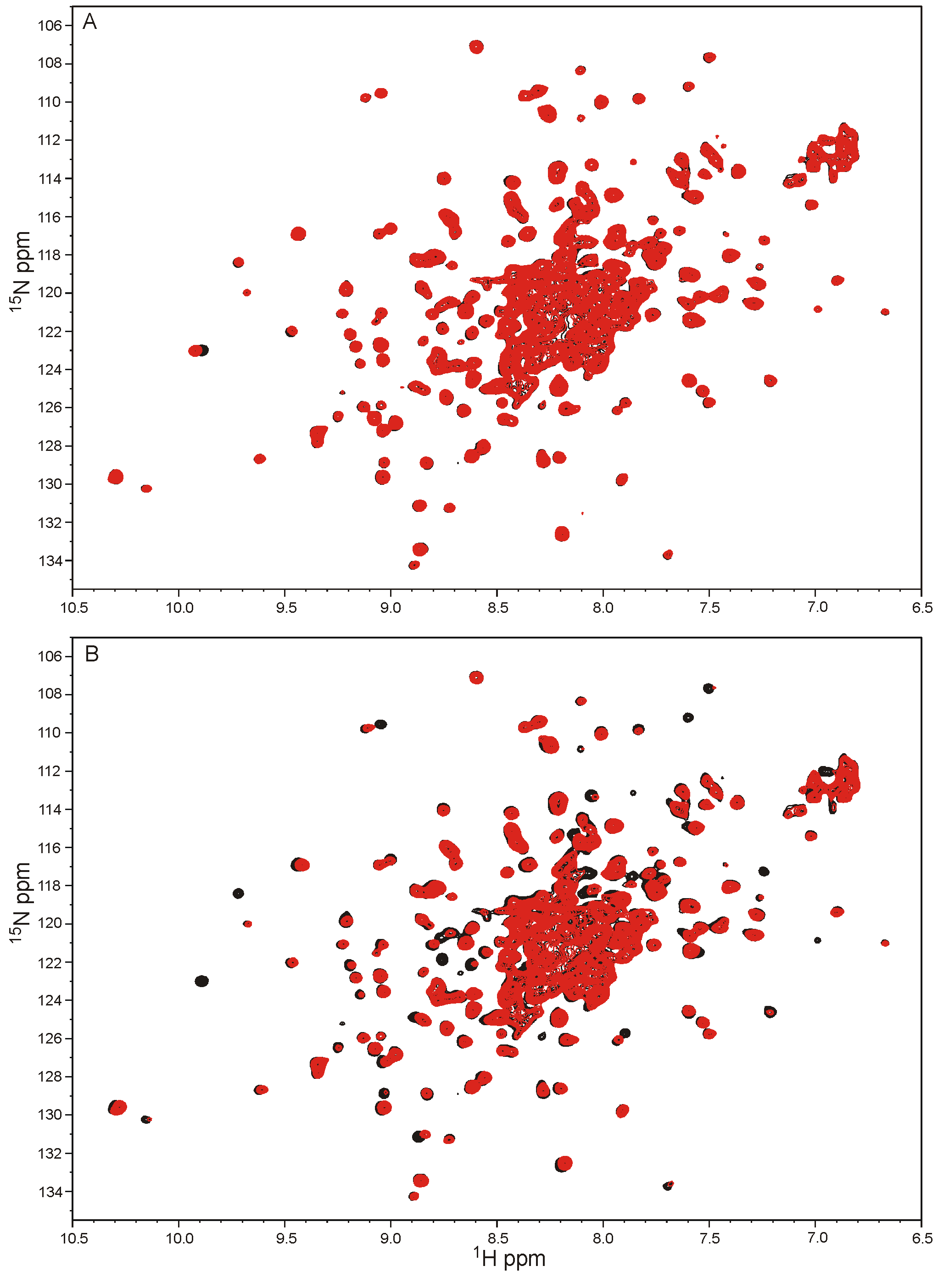

Supplement: Figure S5 — Superposition of 1H-15N-TROSY spectra of dark state YtvA without and with added ligands. 1H-15N-TROSY spectra of (A) 50 µM uniformly 2H-15N-labeled YtvA without (black) and with 500 µM GTP (red) and (B) 50 µM uniformly 2H-15N-labeled YtvA without (black) and with 500 µM BODIPY-GTP (red). All spectra were recorded with YtvA kept in the dark state. (TIF) [file pone.0029201.s005.tif]

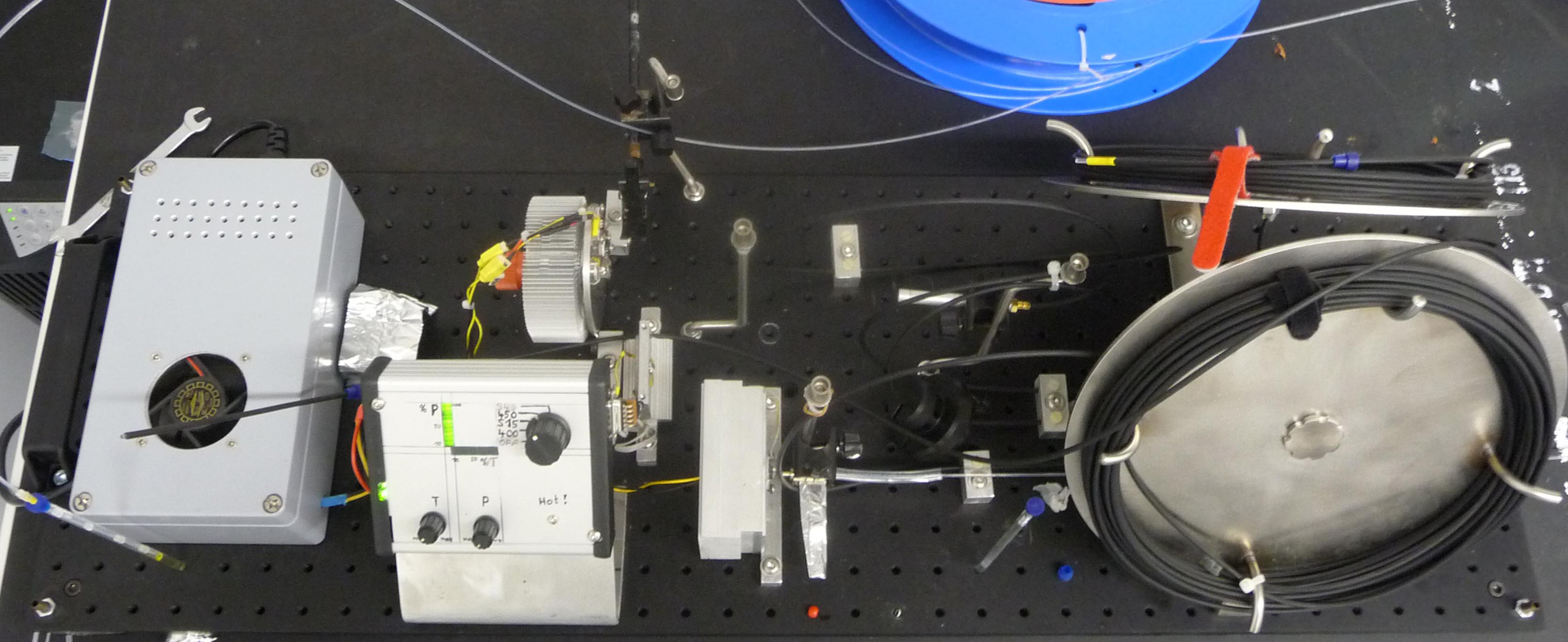

Supplement: Figure S6 — LED based Fiber Coupled Light Source for NMR sample illumination. The light of a modified high power LED (Luxeon LXHL-LR5C, λmax = 455 nm) is butt coupled into an optical fiber (Thorlabs 0,48NA multimode fiber BFH48-1000) and directly transmitted into a 5 mm NMR sample tube. The optical output power is adjustable by means of its intensity and duty cycle from 0 mW up to 8 mW (measured with a S302C Thermal Power Head, Thorlabs). (JPG) [file pone.0029201.s006.jpg]

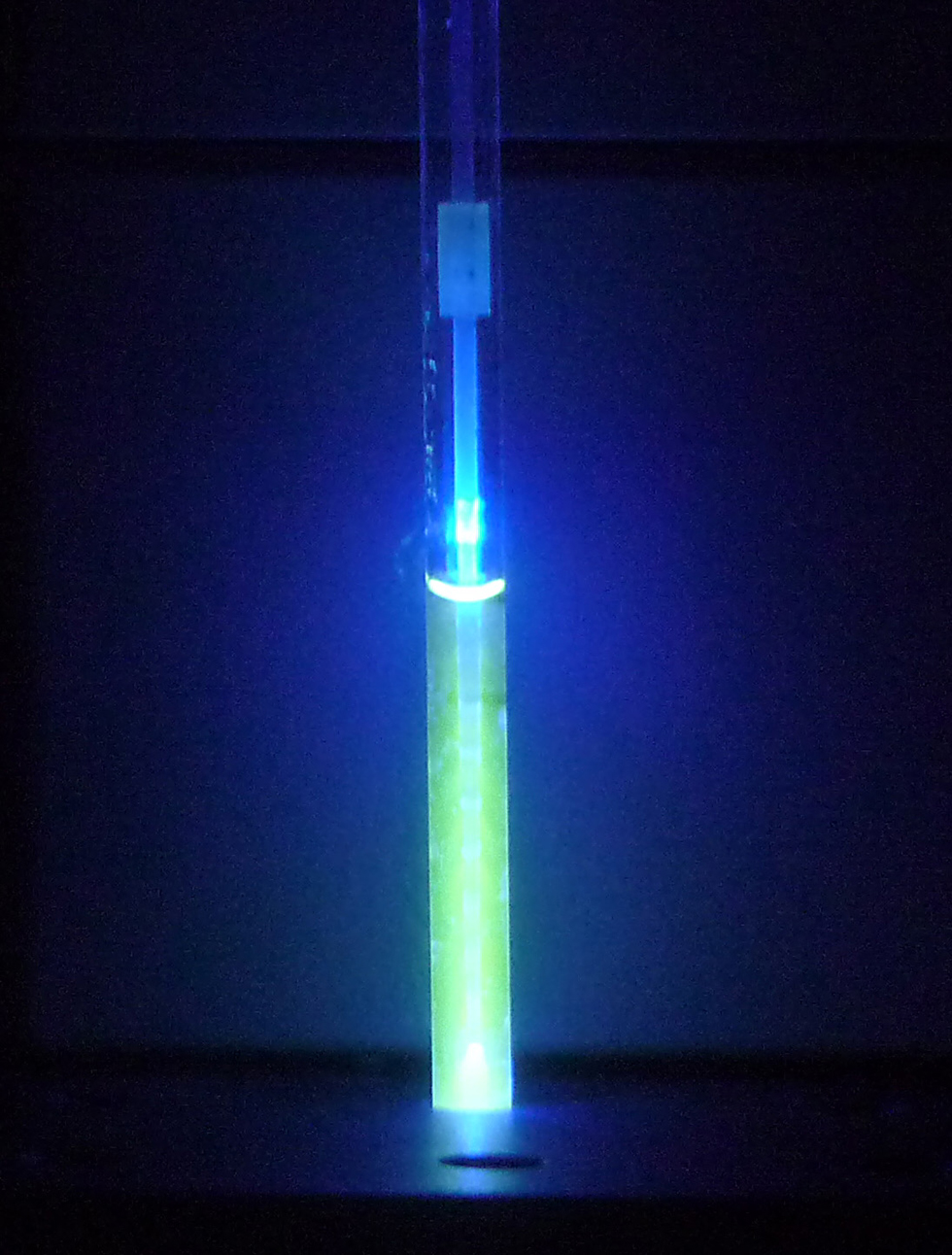

Supplement: Figure S7 — NMR sample illumination with our Fiber Coupled Light Source. Shown is the illumination of YtvA (500 µM in PBS) at λmax = 455 nm directly inside the NMR tube. To achieve a homogeneous illumination within the hole sample the end of the fiber was etched stepwise (12 steps, 3 mm per step) in a mixture containing 30% hydrofluoric acid and 20% sulfuric acid at 60°C as described earlier by Kuprov et al. [44]. (JPG) [file pone.0029201.s007.jpg]
